# Time course of the effect of ferumoxytol on T1-relaxation times of blood, liver, myocardium, and acute infarction

**DOI:** 10.1186/1532-429X-14-S1-P52

**Published:** 2012-02-01

**Authors:** Deneen Spatz, Igor Klem, Lowie M Van Assche, Enn-Ling Chen, Wolfgang G Rehwald, Han W Kim, Christoph J Jensen, Elizabeth Jenista, Raymond J Kim

**Affiliations:** 1 Duke Cardiovascular Magnetic Resonance Center, Durham, NC, USA

## Background

Intravenous iron-supplementation drugs are frequently used for treatment of iron-deficiency anemia in chronic kidney disease. Some iron-agents alter tissue T1-relaxation times (T1) for days after administration, and could obscure MRI diagnosis. Conversely, these agents may have the potential to delineate pathology. We sought to investigate the T1-shortening effect of ferumoxytol and iron-dextran, determine how T1 of blood, liver, and myocardium change over time in-vivo after iron administration, and explore the utility of these agents for imaging acute myocardial infarction (MI).

## Methods

We determined in-vitro T1 of ferumoxytol and iron-dextran (20mg Fe ad 500ml 0.9%NaCl). Seven dogs with acute, reperfused MI were scanned five days later on a 3.0T MRI-scanner, at which time ferumoxytol (n=5) or iron-dextran (n=2) was administered in clinically used doses (approx. 130mg iron). Inversion-recovery, gradient-echo images with various inversion-times (115-1600ms) were acquired prior to and serially after iron-injection at multiple time-points on day 1 (n=7), 2 (n=5), 3 (n=2), and 7 (n=7). T1 was determined by standard curve-fitting.

## Results

In-vitro T1 of ferumoxytol and iron-dextran were 13% and 89% of 0.9%NaCl, respectively. T1 of blood, myocardium, and liver were 2027±421ms, 1384±143ms, and 806±74ms, respectively. Results for ferumoxytol were: T1 of blood dropped to 7% (p<0.001) 29±24min after ferumoxytol-injection, and fully recovered by day 2 in 3/5, and by day 7 in all animals. T1 of liver dropped to 36% (p<0.001) at 29±24min; notably, beyond 2 hours and still present at 1 week, liver-signal was attenuated by T2*-effects, which precluded calculation of T1. T1 of normal myocardium dropped to 51% (p<0.001) at 39.1±21.6min, and completely recovered by day 7 in all animals. Kinetics of ferumoxytol in MI was heterogenous, when T1 of normal myocardium was shortest, T1 of MI was the same or longer in all animals. At 2-5 hours, T1 of MI was shorter than myocardium in 3/5, longer in 1/5 (with no-reflow), and same in 1/5 animals (small MI). Based on differential kinetics of ferumoxytol in MI and normal myocardium, acute MI was visualized at some time-point in all animals. T1 for all tissues were similar before and after iron-dextran (p>0.05).

## Conclusions

Ferumoxytol may affect cardiovascular MR beyond 2 days and liver MR beyond 1 week after administration of doses used clinically for iron-deficiency anemia. Unless recognized, this could affect MRI diagnosis. The differential kinetics suggest a potential use of ferumoxytol for delineation of acute MI.

## Funding

Funded in part by Luitpold Pharmaceuticals, INC.

**Figure 1:**
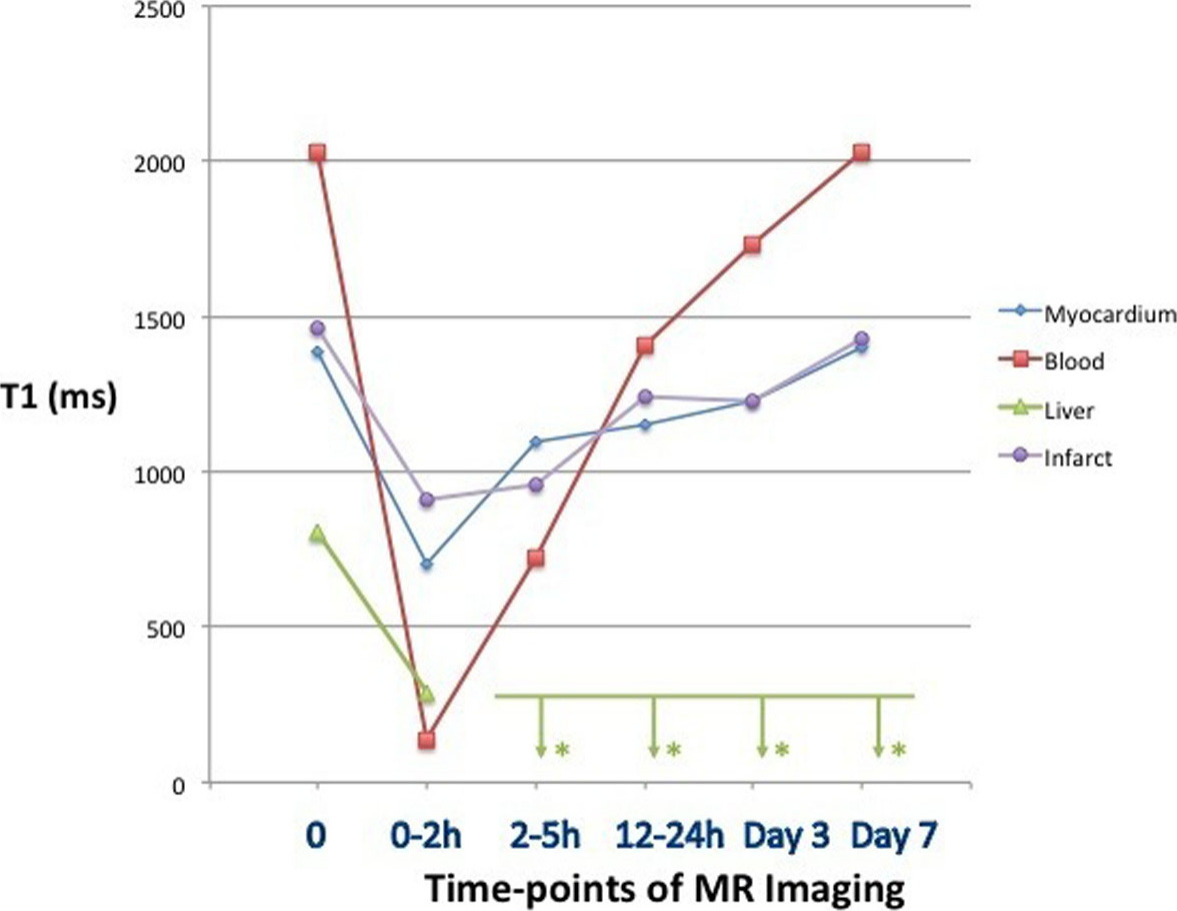
Time course of T1-times in 5 animals, that received ferumoxytol. * T1 of liver is reduced early after ferumoxytol, but after 2 hours, the extent of T1 reduction cannot be determined due to T2*-effects.

**Figure 2:**
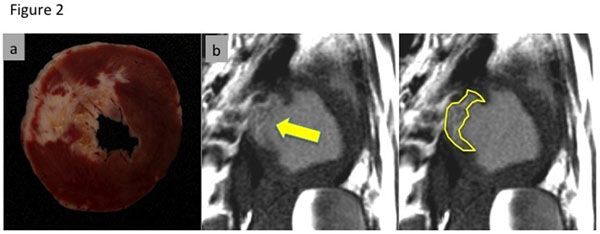
Pathology (a) and delayed enhancement image (b) 4:33 hrs after administration of ferumoxytol in an animal with acute infarction in the LAD (arrow).

